# High Alanine Aminotransaminase Associated with Daptomycin Use in a Premature Infant

**DOI:** 10.34763/jmotherandchild.20212501.d-20-00020

**Published:** 2021-10-11

**Authors:** Suzan Suhail Asfour, Fahad Aljobair, Adli Abdelrahim, Mountasser Mohammad Al-Mouqdad

**Affiliations:** 1 Clinical Pharmacy Department, Pharmacy Department, King Saud Medical City, Riyadh, Saudi Arabia; 2 Pediatric Infectious Disease Department, Hospital of Pediatric, King Saud Medical City, Riyadh, Saudi Arabia; 3 Neonatal Intensive Care Unit, Hospital of Pediatric, King Saud Medical City, Riyadh, Saudi Arabia

**Keywords:** coagulase-negative staphylococci, daptomycin, disseminated intravascular coagulation, hepatotoxicity, infant

## Abstract

Daptomycin is a cyclic lipopeptide antibiotic, a fermented product derived from *Streptomyces roseosporus* that is active against gram-positive bacteria. We report on a premature infant who developed hepatotoxicity as an adverse drug reaction after the administration of daptomycin 6 mg per kg per dose every 12 h. The patient had an unexpectedly sharp rise of alanine aminotransaminase, prothrombin time and international normalised ratio on the second day following daptomycin administration. This case illustrates a previously unrecognised adverse drug effect associated with daptomycin use in infants.

## Introduction

Gram-positive organisms including coagulase-negative staphylococci are the most common causative organisms of late-onset sepsis in infants [1]. Daptomycin is a cyclic lipopeptide antibiotic that provides an activity against gram-positive pathogens, including vancomycin-resistant organisms [2]. Daptomycin has been approved for the treatment of complicated skin and skin structure infections and bacteraemia caused by *Staphylococcus aureus* in adults and in paediatrics older than one year [3]. Preliminary data provided the safety and efficacy profile of daptomycin use in infants with mild adverse drug reactions (ADRs). The most common reported ADRs were phlebitis, infusion site reactions and elevation in liver enzymes and creatine phosphokinase (CPK) [4,5]. We report the first case of a preterm infant who developed severe hepatotoxicity associated with disseminated intravascular coagulation (DIC) following intravenous administration of daptomycin for a persistent infection caused by coagulase-negative staphylococci.

## Case report

A 25-week male infant with a birth weight of 750 g was delivered by an emergency caesarean section to a 40-year-old mother. There was no evidence of chorioamnionitis. A course of steroid had been given. His Apgar score was 2 and 5 at 1 and 5 minutes, respectively. Soon after delivery, the baby was transferred to the neonatal intensive care unit, received surfactant and was connected to a mechanical ventilator. An umbilical venous catheter was inserted, a blood culture sent and antibiotics with ampicillin and gentamicin were started. Later, a brain ultrasound was done and showed grade II of intraventricular haemorrhage. The transthoracic echocardiogram was also done on the fifth day of life, showing large patent ductus arteriosus. Thus, one course of ibuprofen was given. At the age of seven days, antibiotics were changed to vancomycin and cefotaxime. At ten days of life, the baby developed a perforated jejunum. The blood culture result came back positive with *Staphylococcus haemolyticus* that was sensitive to vancomycin, trimethoprim/ sulfamethoxazole, rifampicin and linezolid, while it was resistant to oxacillin. Thus, cefotaxime was discontinued. Despite the appropriate dose and appropriate serum drug level of vancomycin and rifampicin, the blood culture remained positive. In addition, the vancomycin minimum inhibitory concentration increased to 2.0 μg/ml instead of 1.0 μg/ml. Thus, at the age of 50 days, vancomycin was changed to linezolid. At the same time, levofloxacin was started due to a Stenotrophomonas maltophilia infection from bronchial wash and ineffective treatment with cotrimoxazole. At the age of 55 days and due to failure response with linezolid, daptomycin was initiated with a continuation of levofloxacin. Before initiation of intravenous daptomycin, the patient’s serum alanine aminotransaminase (ALT) was 101.2 U/L, the serum aspartate aminotransaminase (AST) was 440.8 U/L, the total bilirubin was 509.8 μmol/L and the direct bilirubin was 415.6 μmol/L. On day two of daptomycin administration, laboratory results indicated severe hepatotoxicity and DIC: ALT was 1144 U/L, AST was 1115 U/L, the total bilirubin was 616.3 μmol/L and the direct bilirubin was 443 μmol/L.

**Figure 1:**
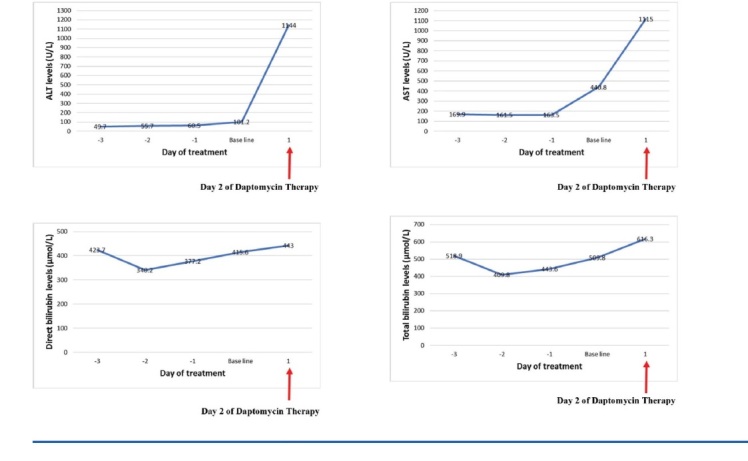
A graph showing the trend in selected liver function tests before and after the initiation of daptomycin therapy. ALT, alanine aminotransferase; AST, aspartate aminotransferase.

Additionally, the prothrombin time (PT) was 56.1 seconds, the international normalised ratio (INR) was 6.19 and the partial thromboplastin time was 169.9 seconds. Written inform consent was obtained from family. To investigate other potential causes of liver damage, a urine culture for cytomegalovirus was performed, and the result was negative. Additionally, an abdominal ultrasound was done, and the result documented normal hepatic anatomy. The patient died with 48 hours of daptomycin initiation.

## Discussion

The above case is the first that enlightens the possible rare and serious ADR associated with daptomycin therapy in infants. Daptomycin is active against gram-positive bacteria [2]. The mechanism of action involves binding to bacterial membranes causing depolarisation. As a result, a rapid inhibition of protein, DNA, and RNA synthesis results in bacterial cell death [6]. Pharmacokinetics and pharmacodynamics studies of daptomycin in infant populations have not been studied extensively. Thus, data are still limited on the optimum dosing regimen of daptomycin in infants [7,8]. Bradley et al. conducted a single dose pharmacokinetics study of daptomycin in 19 infants aged between 3 months and 24 months [7]. Cohen-Wolkowiez et al. conducted the same study in infants younger than 120 days. [8]. Preliminary results showed an inverse linear correlation exists between clearance of daptomycin and age [4,7,8]. Daptomycin elimination occurs primarily through the renal system, with 50% of the administered dose excreted within 24 h as unchanged drug. Neonates and infants had higher clearance rates compared with adults and adolescents. The mean clearance rate is 21 mL per h per kg in neonates and 11 mL per h per kg in children [4]. This can explain why higher doses are needed to achieve the warranted therapeutic drug concentration in infants and younger children [9]. The available mounting literature indicate that daptomycin is well tolerated in infants. Common ADRs, including mild CPK and transaminase increase, headache, phlebitis, and local reactions, are reported in comparable or lower rates compared with adults’ series [4,5]. Animal data on neonatal dogs showed serious adverse efects on muscular, neuromuscular and/or nervous systems. As a result, daptomycin is not approved for use in neonates and infants younger than one year [3]. In addition, the product labels an elevation of PT and INR as rare and uncommon ADRs, respectively [3]. Our case initially received an appropriate therapy with vancomycin plus rifampin. However, *Staphylococcus hemolyticus* became protracted and resistant even to linezolid, necessitating the use of daptomycin therapy that is still not yet approved for use by the European Medicines Agency in this age group. Our patient experienced an elevation of ALT, AST, PT, INR and PTT on the second day following the administration of intravenous daptomycin 6 mg per kg per dose every 12 h. Using the Naranjo probability scale for this patient’s adverse efect indicates a probable relationship between hepatotoxicity with DIC and daptomycin. When the medications chart was reviewed, we found two possible causes for hepatotoxicity: daptomycin and levofloxacin. Since the patient was started on levofloxacin several days before, we excluded levofloxacin. In addition, Nicole Bohm et al. did a retrospective study where they found that most cases showed increases in ALT, bilirubin or both ALT and bilirubin on the second day of daptomycin administration [10]. Daptomycin-induced serious hepatotoxicity associated with DIC appears to be extremely rare. To our knowledge, only a few cases of mild, nonfatal hepatoxicity have been reported in paediatrics and infants. Health care practitioners should be aware of these complications, which require closely monitoring hepatic function. Daptomycin should be promptly discontinued once serious and unusual ADRs are suspected, with further investigation to reduce the incidence of morbidity and mortality.

## Conclusions

Although daptomycin appears to be an effective and safe alternative therapy in infants with invasive gram-positive infection, our case highlights that foetal hepatotoxicity associated with DIC can be observed with the administration of intravenous daptomycin at a dose of 6 mg per kg every 12 h in infants. Further studies are needed to gain knowledge about the pharmacokinetics, pharmacodynamics, as well as safety and efficacy of this drug in infants.

## References

[1] Stoll BJ, Hansen N, Fanaroff AA, Wright LL, Carlo WA, Ehrenkranz RA (2002). Late-onset sepsis in very low birth weight neonates: the experience of the NICHD Neonatal Research Network. Pediatrics.

[2] Critchley IA, Draghi DC, Sahm DF, Thornsberry C, Jones ME, Karlowsky JA (2003). Activity of daptomycin against susceptible and multidrug-resistant gram-positive pathogens collected in the SECURE study (Europe) during 2000-2001. J Antimicrob Chemother.

[3] Cubicin (daptomycin for injection) [package insert].

[4] Cohen-Wolkowiez M, KM Watt, CP Hornik, DK Jr Benjamin, PB Smith (2012). Pharmacokinetics and tolerability of single-dose daptomycin in young infants. Pediatr Infect Dis J.

[5] Abdel-Rahman SM, Chandorkar G, Akins RL, Bradley JS, Jacobs RF, Donovan J (2011). Single-dose pharmacokinetics and tolerability of daptomycin 8 to 10 mg/kg in children aged 2 to 6 years with suspected or proved gram-positive infections. Pediatr Infect Dis J.

[6] Sader HS, Farrell DJ, Flamm RK, Jones RN (2014). Daptomycin activity tested against 164457 bacterial isolates from hospitalised patients: summary of 8 years of a Worldwide Surveillance Programme (2005-2012). Int J Antimicrob Agents.

[7] Bradley JS, Benziger D, Bokesch P, Jacobs R (2014). Single-dose pharmacokinetics of daptomycin in pediatric patients 3-24 months of age. Pediatr Infect Dis J.

[8] Cohen-Wolkowiez M, PB Smith, DK Jr Benjamin, VG Jr Fowler, KC Wade (2008). Daptomycin use in infants: report of two cases with peak and trough drug concentrations. J Perinatol.

[9] Abdel-Rahman SM, DP Benziger, RF Jacobs, HS Jafri, EF Hong, GL Kearns (2008). Single-dose pharmacokinetics of daptomycin in children with suspected or proved gram-positive infections. Pediatr Infect Dis J.

[10] Bohm N, Makowski C, Machado M, Davie A, Seabrook N, Wheless L (2014). Case report and cohort analysis of drug-induced liver injury associated with daptomycin. Antimicrob Agents Chemother.

